# Differential Diagnosis

**Published:** 1888-04

**Authors:** 


DIFFERENTIAL DIAGNOSIS. A Manual of the Comparative Semeiology of the more important Diseases. By F. DeHaviland Hall, M. D., Assistant Physician to the Westminster Hospital, London. Philadelphia: D. G. Brinton.

This is the third American edition of this popular work. It has undergone thorough revision by Dr. Frank Woodbury, and many additions of a practical nature have been made. There is no doubt but that it will find as much favor in the eyes of the profession as the two previous editions.

